# Preliminary spatiotemporal analysis of the association between socio-environmental factors and suicide

**DOI:** 10.1186/1476-069X-8-46

**Published:** 2009-10-01

**Authors:** Xin Qi, Shilu Tong, Wenbiao Hu

**Affiliations:** 1 School of Public Health, and Institute of Health and Biomedical Innovation, Queensland University of Technology, Kelvin Grove, Queensland 4059, Australia; 2 School of Population Health, University of Queensland, Herston, Queensland 4006, Australia

## Abstract

**Background:**

The seasonality of suicide has long been recognised. However, little is known about the relative importance of socio-environmental factors in the occurrence of suicide in different geographical areas. This study examined the association of climate, socioeconomic and demographic factors with suicide in Queensland, Australia, using a spatiotemporal approach.

**Methods:**

Seasonal data on suicide, demographic variables and socioeconomic indexes for areas in each Local Government Area (LGA) between 1999 and 2003 were acquired from the Australian Bureau of Statistics. Climate data were supplied by the Australian Bureau of Meteorology. A multivariable generalized estimating equation model was used to examine the impact of socio-environmental factors on suicide.

**Results:**

The preliminary data analyses show that far north Queensland had the highest suicide incidence (e.g., Cook and Mornington Shires), while the south-western areas had the lowest incidence (e.g., Barcoo and Bauhinia Shires) in all the seasons. Maximum temperature, unemployment rate, the proportion of Indigenous population and the proportion of population with low individual income were statistically significantly and positively associated with suicide. There were weaker but not significant associations for other variables.

**Conclusion:**

Maximum temperature, the proportion of Indigenous population and unemployment rate appeared to be major determinants of suicide at a LGA level in Queensland.

## Background

Suicide is one of the major causes of mortality around the world with about 877,000 suicide deaths each year globally [1]. Socio-environmental impacts on mental health, including suicide, have drawn increasing research attention, especially in recent years as global socio-environmental conditions change rapidly [2,3].

A number of studies have examined the impact of meteorological factors on suicide and found that lower suicide rates were associated with increased rainfall [4], decreased temperature [5], decreased humidity [6], and increased sunshine [7]. Additionally, some studies indicated that suicide rates varied with season [8,9]. Socioeconomic status [10,11], unemployment rate [12-14], country of birth [15,16], governmental policy [17,18] and intervention [19,20] were also associated with suicide in different countries and areas.

Most of the previous suicide studies have focused on either meteorological or socioeconomic factors alone, and none has examined their combined effect. As all these factors can influence suicide in different aspects, the impact of these factors on suicide, thus, should be studied in a systematic way, to help formulating effective suicide prevention strategies. In addition, few of the previous studies have applied geographical information system (GIS) and or spatial analysis approaches to assess the geographical difference of suicide, and the socio-environmental impact on suicide [21,22].

**Figure 1:**
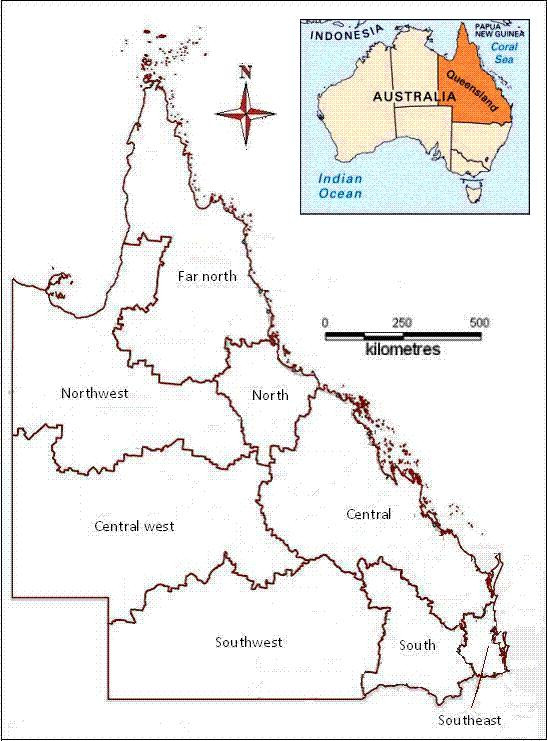
The regions in Queensland

This study examined the association of socio-environmental determinants with suicide in Queensland, Australia using a GIS-based ecological study design. Queensland is the second largest states in Australia and it lies on the northeast of the continent, covering an area about 1.73 million km^2^, with a total population about 4.18 million in June 2007. Southeast Queensland (SEQ) accounts for less than 1.3% of total area, but had 65.4% of total population [23]. Other places, especially the inland areas, have much less population density than that of the whole state. The whole state is divided into a few regions as Figure 1. The climate conditions vary across the whole states. The far north and coastal areas are hot and humid in summer, while the highlands near coast and south-eastern coasts are warm and humid in summer. The inland areas in the southeast have temperate or warm summer, but cold winter. The western areas of Queensland have hot and dry summers, and mild and cold winters [24]. Queensland had more rapid increase in economy than the rest areas of Australia between 1992 until now, except for the financial year 1995-1996 [25]. The major industries in Queensland are agriculture, mining, financial services and tourism.

## Methods

### Study design

Spatiotemporal analysis of the impact of socio-environmental factors on suicide is critical because the distribution of suicide deaths and its determinants may vary with time and place, especially in Queensland, a large state with a wide range of climatic conditions and socioeconomic positions. The study consisted of four phases: data collection, data linkage and management, descriptive analyses, bivariable and multivariable analyses.

### Data sources

The meteorological data, including monthly rainfall (RF), maximum temperature (MaxT) and minimum temperature (MinT) were supplied by the Australian Bureau of Meteorology. Suicide, socioeconomic and demographic data were obtained from the Australian Bureau of Statistics (ABS).

The suicide data, covering a five-year period (1999-2003), included information on age, sex, year and month of suicide and Statistical Local Area (SLA) code. There are 125 Local Government Areas (LGAs) in Queensland in 2001 and each LGA has one or more SLAs. Suicide data were transferred into the LGA-based data, using the LGA codes from Australian Standard Geographical Classification (ASGC) [26]. Average suicide counts in total and by gender were calculated for each season at the LGA level (September, October and November for spring; December, January and February for summer; March, April and May for autumn; and June, July and August for winter).

The meteorological database was composed of monthly grid (0.25°*0.25°, longitude and latitude; equivalent to the area of about 25 km*25 km) data. We used Vertical Mapper, a GIS tool, to transfer the meteorological data into the LGA data. Vertical Mapper was incorporated into the MapInfo, which was then used as a platform to perform the data link, data transfer and spatial display. After primary data retrieving and transferring, the structure of monthly meteorological data at the LGA level was established. The means of seasonal meteorological data at LGA level were calculated from monthly data.

Socio-economic Indexes for Area (SEIFA) and demographic data at the LGA level were based on CDATA 2001 of ABS, a database which provides information of 2001 Australian Census of Population and Housing, digital statistical boundaries, base map data and socio-economic data. We directly applied SEIFA and demographic data from the CDATA in the analysis.

SEIFA included four indices: the Index of Relative Socio-economic Advantage and Disadvantage (i.e., IRSAD, the higher IRSAD index, the higher socioeconomic position), the Index of Relative Socio-economic Disadvantage (i.e., IRSD, reflecting disadvantage such as low income and education level, high unemployment and unskilled occupations), the Index of Economic Resources (i.e., IER, reflecting the general level of availability to economic resources of residents and households) and the Index of Education and Occupation (i.e., IEO, reflecting the general educational level and occupational skills of people). All these indices were obtained from CDATA 2001.

Demographic variables included population, Indigenous population, unemployed population, population with low individual income (below AU$ 200 per week) and low education level (Year 9 and below). Using these numbers the following statistics were calculated: the proportion of Indigenous population (PIP), unemployment rate (UER), proportion of population with low individual income (PPLII) and proportion of population with low education level (PPLEL).

### Data analyses

A series of GIS and statistical methods were used to analyse these data. MapInfo (including Vertical Mapper incorporated) was used to explore the spatial patterns of socio-environmental variables and suicide.

Univariable analysis was applied to describe characteristics of each variable (suicide and socio-environmental factors). This step is important because it can show the pattern of distribution of each variable, and then select appropriate approaches for bivariable and multivariable analysis. Pearson correlations were applied for bivariable analysis after some non-normally-distributed data (suicide mortality rate, rainfall, IRSD, PIP and UER) were transformed into approximately normally-distributed values by logarithm transformation. The multicollinearity was tested for selecting variables for the multivariable modelling process. The multivariable generalized estimating equation (GEE) regression models with a Poisson link were developed to assess the possible impact of socio-environmental factors on suicide, after adjustment for the effects of potential confounders. The GEE model is well suited to analyse the repeated longitudinal data (e.g., climate data) [27]. This approach has also been used in other studies [28,29]. Spatial autocorrelation is defined as an auto-correlated association of a certain spatial variable with its spatial location, which means observations have similar values if they are close to each other in geographical aspect [30]. In this study, spatial autocorrelation test was applied to examine the variation of suicide between small areas. Semivariogram analysis was used to explore the spatial structure and spatial autocorrelation of suicide mortalities in Queensland, where semivariogram values were calculated on the basis of residuals. If there is spatial autocorrelation in model residuals, values are typically low and the semivariance increases with separation distance [30,31]. Statistical Package for the Social Sciences (SPSS) and S+ SpatialStats software were used for data analysis.

## Results

### Univariable analysis

Table 1 demonstrates the distribution of suicide in Queensland between 1999 and 2003 by gender, year and month. There were 2,445 suicide cases in Queensland, with 1,957 males (80.0%) and 488 females (20.0%). There was no significant difference in monthly variation of suicide by gender.

**Table 1:** Suicide counts by year and month for both male (upper value) and females (lower value, italics)*

Years	**Jan**.	**Feb**.	**Mar**.	**Apr**.	May	**Jun**.	**Jul**.	**Aug**.	**Sep**.	**Oct**.	**Nov**.	**Dec**.
1999	22 *7*	32 *7*	27 *3*	30 *6*	25 *9*	24 *3*	38 *12*	47 *10*	31 *5*	40 *10*	30 *7*	32 *10*
2000	37 *12*	43 *7*	42 *8*	28 *9*	37 *14*	37 *10*	26 *9*	34 *9*	29 *11*	51 *10*	36 *7*	44 *9*
2001	43 *7*	28 *6*	36 *15*	36 *7*	33 *9*	31 *8*	31 *4*	33 *13*	29 *7*	27 *11*	24 *2*	38 *12*
2002	43 *11*	31 *9*	41 *8*	33 *6*	31 *8*	32 *10*	38 *7*	32 *10*	29 *7*	36 *8*	34 *8*	41 *8*
2003	37 *10*	30 *9*	24 *3*	21 *10*	11 *5*	17 *5*	26 *11*	44 *6*	35 *8*	38 *6*	31 *7*	11 *3*
Total	182 *47*	164 *38*	170 *37*	148 *38*	137 *45*	141 *36*	159 *43*	190 *48*	153 *38*	192 *45*	155 *31*	166 *42*

In the population of the whole Queensland, 36.4% of males and 34.4% of females were 24-year age and below. 29.5% of males and 30.1% of females aged between 25 and 44. 23.5% of male population and 22.9% of female population were between 45 and 64-year-age. Other people aged at 65-year and above. In the age structure of suicides, 16.4% of males and 15.2% females were adolescents and youth (aged at 24-year or below). 46.9% of males and 48.6% of females aged between 25 and 44-year. 24.6% of male suicides and 25.8% of female suicides were between 45 and 64-year age. 12.1% of males and 10.4% of females were older-aged adults (65 and over).

Table 2 shows the characteristics of each variable (suicide mortality rate and socio-environmental variables) at the LGA level over seasons. It shows a range of variation for each variable. Some data are normally-distributed (MaxT, MinT, IRSAD, IER, IEO, PPLII in total and by gender, PPLEL in total and by gender). Some other variables are non-normally-distributed (suicide mortality rate, suicide ASM by gender, rainfall, IRSD, PIP in total and by gender and UER in total and by gender).

**Table 2:** Characteristics of suicide mortality, socio-demographic and environmental factors*

	Mean	SD	Minimum	Percentiles			Maximum
					
				25	50	75	
Total mortality (per 100,000)	4.28	15.038	0.00	0.00	0.00	3.17	211.64
Male ASM rate (per 100,000)	6.96	25.872	0.00	0.00	0.00	3.94	410.68
Female ASM rate (per 100,000)	1.34	10.053	0.00	0.00	0.00	0.00	225.73
RF (mm)	195.0	201.53	0.1	76.5	143.8	237.8	1865.4
MinT (°C)	15.1	5.08	2.2	12.1	15.6	18.7	26.0
MaxT (°C)	28.0	4.36	16.7	25.0	28.2	30.9	39.1
IRSAD	935.61	41.476	831.36	910.32	930.64	962.72	1059.84
IRSD	957.71	69.305	472.08	946.32	972.48	992.40	1048.88
IER	942.25	52.481	835.52	903.68	939.36	975.44	1083.76
IEO	929.05	35.566	815.68	909.60	925.84	945.52	1064.32
Total PIP (%)	7.79	14.392	0.00	1.92	2.86	6.15	87.51
Male PIP (%)	7.43	13.938	0.00	1.88	2.98	5.73	86.65
Female PIP (%)	8.23	14.987	0.00	1.89	3.14	6.61	88.67
Total UER (%)	6.76	3.819	0.00	4.03	6.04	8.76	23.25
Male UER (%)	7.14	4.526	0.00	4.06	6.45	9.36	26.71
Female UER (%)	6.25	3.110	0.00	4.16	6.11	7.93	18.37
Total PPLII (%)	28.14	7.472	10.65	24.39	27.91	31.77	61.71
Male PPLII (%)	23.06	8.937	4.30	17.66	22.44	27.44	62.22
Female PPLII (%)	34.19	6.611	17.13	30.51	33.63	37.86	65.18
Total PPLEL (%)	22.75	5.468	11.91	19.21	22.94	26.10	47.41
Male PPLEL (%)	24.61	6.234	11.21	20.00	25.25	28.53	50.83
Female PPLEL (%)	20.64	4.992	9.01	17.25	20.70	23.27	43.91

*Note: ASM (age-standard mortality rates); RF (rainfall); MinT (minimum temperature); MaxT (max temperature); IRSAD (Index of Relative Socio-economic Advantage and Disadvantage); IRSD (Index of Relative Socio-economic Advantage and Disadvantage); IER (Index of Economic Resources); IEO (Index of Education and Occupation); PIP (proportion of Indigenous population); UER (unemployment rate); PPLII (proportion of population with low individual income); PPLEL (proportion of population with low educational level).

**Figure 2:**
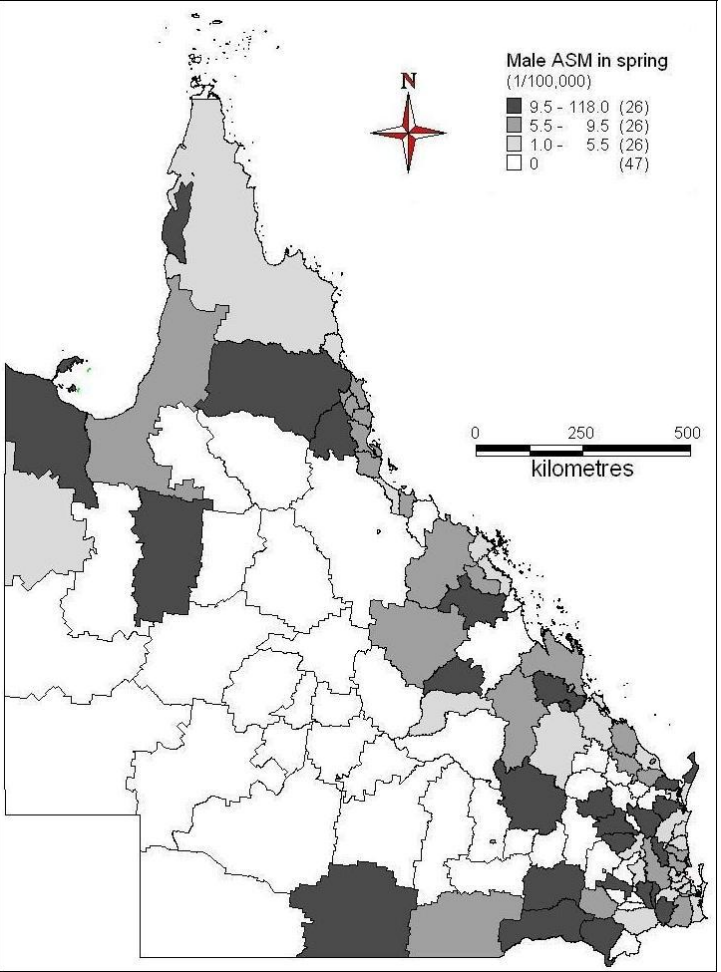
Average male ASM in spring (1999-2003)

**Figure 3:**
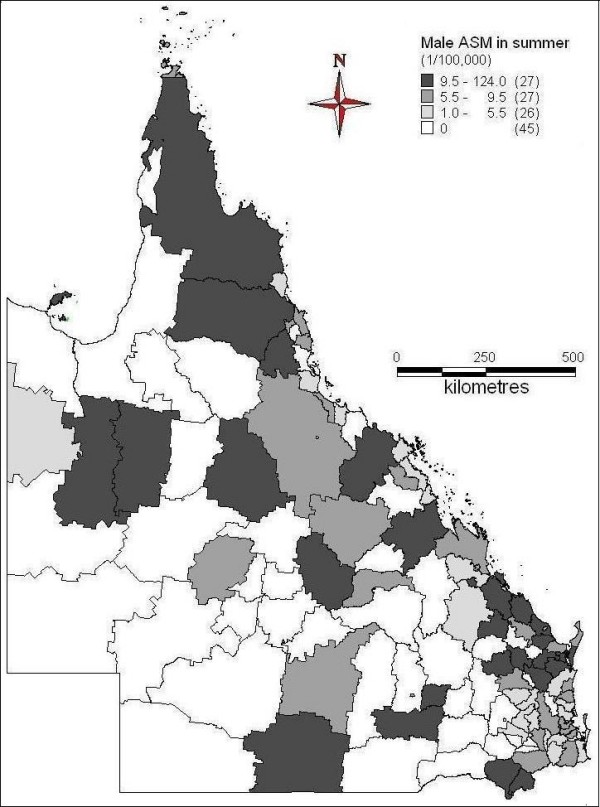
Average male ASM in summer (1999-2003)

**Figure 4:**
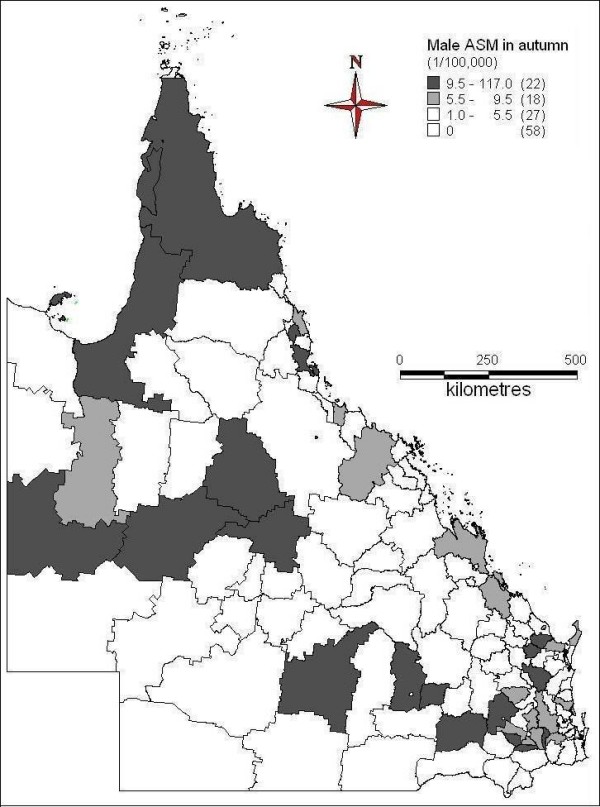
Average male ASM in autumn (1999-2003)

**Figure 5:**
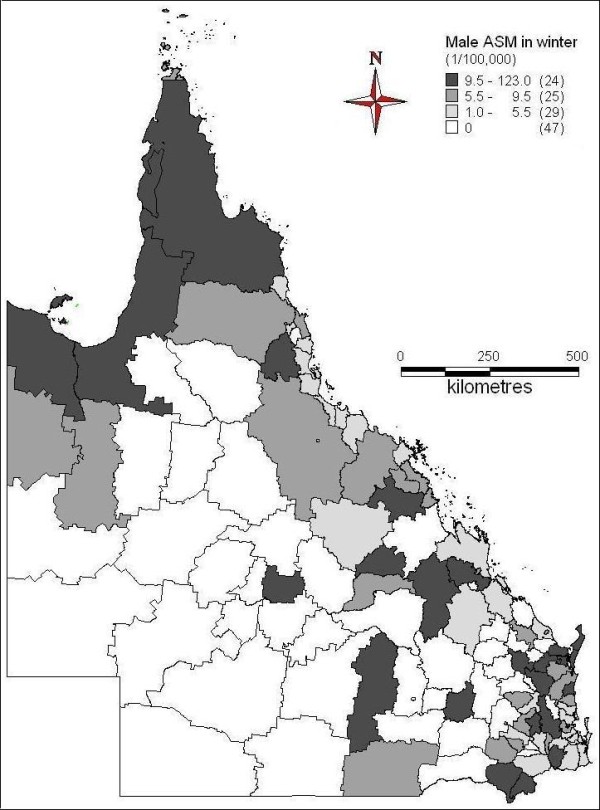
Average male ASM in winter (1999-2003)

Figures 2 to 5 demonstrated the spatial patterns of age-adjusted standard mortality (ASM) of male suicide in Queensland between different seasons. In spring, some of far north, northwest, south, some of southeast and central coast areas had higher suicide ASM, while the inland and south western areas had lower suicide ASM or no suicide record (Figure 2). During the summer time, far north, some of north west, southeast and coastal and some of the south areas had higher suicide ASM; southwest and some of the central south areas had lower suicide ASM or no suicide record (Figure 3). Figure 4 indicates the suicide ASM distribution in autumn. Far north, west, central, some of the coastal and southeast areas had higher suicide ASM; south, southwest and some of the central areas had lower suicide ASM or no suicide record. In winter, some of far north areas, northwest, some of the southeast and east areas had higher suicide mortality rate, while central, southwest and other areas had lower suicide mortality rate or even no suicide record (Figure 5).

**Figure 6:**
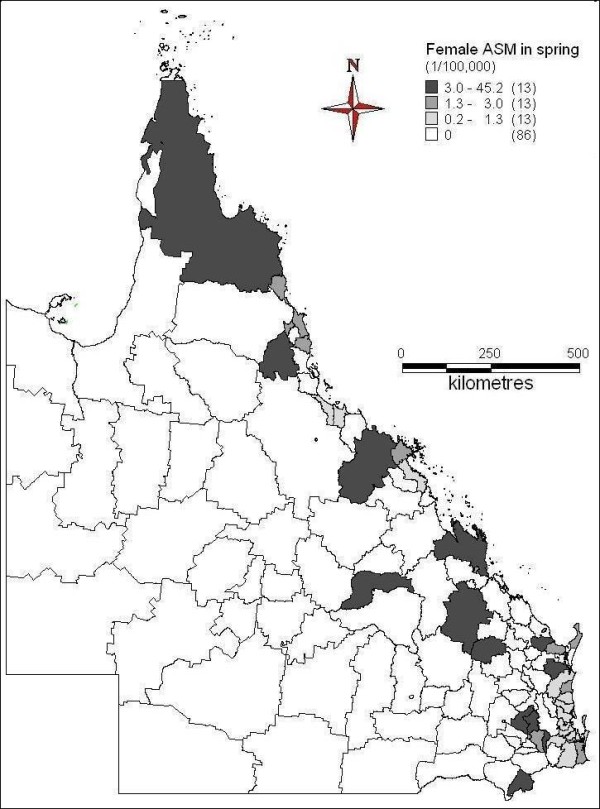
Average female ASM in spring (1999-2003)

**Figure 7:**
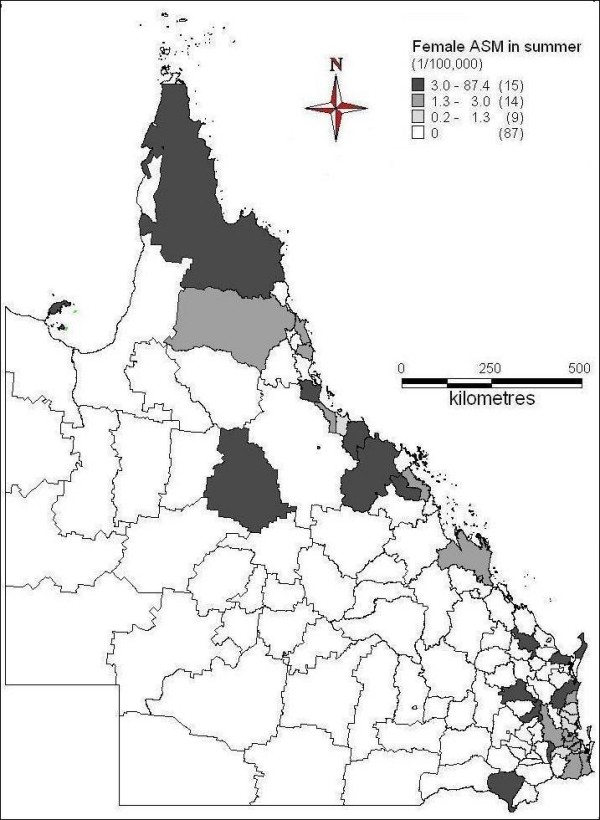
Average female ASM in summer (1999-2003)

**Figure 8:**
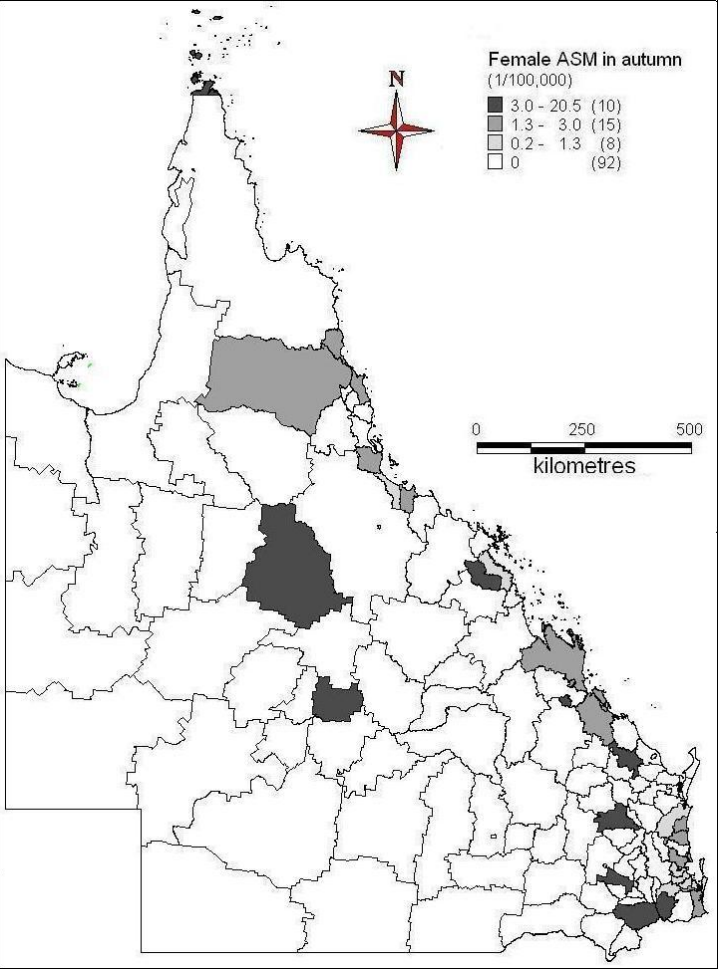
Average female ASM in autumn (1999-2003)

**Figure 9:**
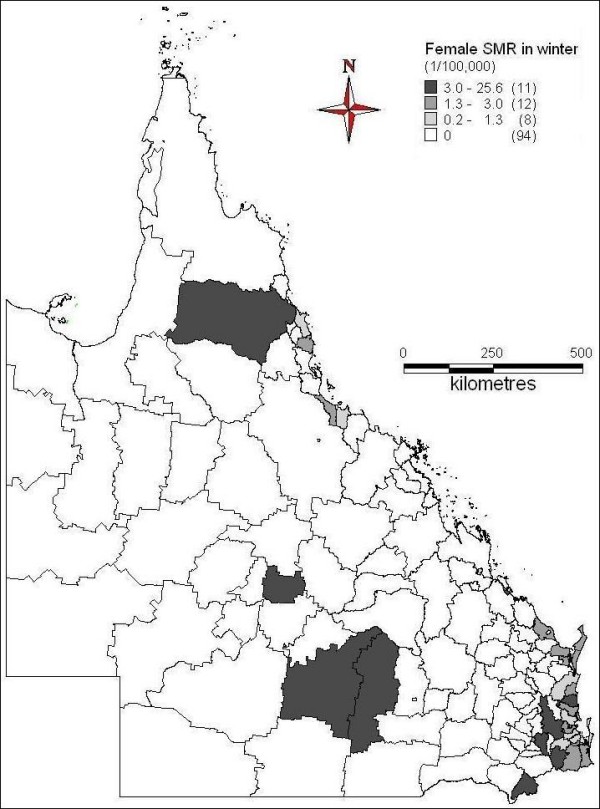
Average female ASM in winter (1999-2003)

The spatial patterns of ASM of female suicide across seasons were indicated in Figures 6 to 9. In spring, far north, north and central coast and some inland areas in the east and south had higher suicide ASM (Figure 6). There was higher suicide ASM in far north, some of north-western areas, some coastal and inland areas in the north and southeast than other areas (Figure 7). In autumn, some areas of central inland and coast, and southeast had higher suicide ASM compared with lower suicide ASM or no suicide record in other areas (Figure 8). There was higher suicide ASM in some parts of far north, central inland and south-eastern areas than other areas (Figure 9). 68.8% to 76.8% of LGAs had no female suicide record within each seasons.

In general, male suicides were recorded in the most LGAs, except for the southwest and some part of the central areas. Only 17 LGAs (13.6% of total) had no suicide recorded in the whole study period. Most female suicides occurred in the southeast, coastal and far north areas. There were very few female suicides in the majority of inland areas Queensland. Almost half (61 of 125) of total LGAs had no female suicide between 1999 and 2003.

### Bivariable analysis

Table 3 demonstrates the correlations between socio-environmental variables and suicide ASM by gender. In males, MinT, MaxT, PIP, PPLII and PPLEL were significantly and positively associated with suicide ASM, while there were negative associations between SEIFA and suicide. However, UER and RF were not significantly associated with male suicide. In females, RF was significantly and positively associated with suicide. SEIFA variables had significant and negative association with suicide except for IEO. PIP, PPLII and PPLEL were significantly and positively associated with suicide. UER (p = 0.062), MinT (p = 0.088) and IEO (p = 0.087) were marginally associated with suicide. However, MaxT (p = 0.502) was not significantly associated with suicide in females.

**Table 3:** Pearson correlations between socio-environmental factors and suicide for both male (upper value) and females (lower value, italics)^§^

	ASM	RF	MinT	MaxT	IRSAD	IRSD	IER	IEO	PIP	UER	PPLII
RF	0.017 0.053**	1.000									
MinT	0.073** 0.034	0.449**	1.000								
MaxT	0.076** 0.013	0.163**	0.848**	1.000							
IRSAD	-0.147** -0.061**	-0.007	0.029	0.017	1.000						
IRSD	-0.296** -0.094**	-0.125**	-0.233**	-0.196**	0.606**	1.000					
IER	-0.091** -0.055**	-0.022	0.104**	0.108**	0.901**	0.361**	1.000				
IEO	-0.141** -0.034	0.090**	-0.053**	-0.140**	0.772**	0.628**	0.445**	1.000			
PIP	0.163** 0.069**	0.091** 0.075**	0.257** 0.250**	0.280** 0.282**	-0.207** -0.189**	-0.603** -0.593**	-0.047* -0.015	-0.277** -0.288**	1.000		
UER	0.001 *0.037*	0.133** 0.115**	-0.019 *-0.018*	-0.221** -0.219**	-0.387** -0.310**	-0.150** -0.155**	-0.434** -0.314**	-0.067** -0.084**	-0.066** -0.107**	1.000	
PPLII	0.232** 0.059**	0.120** 0.015	-0.019 *-0.026*	-0.122** -0.067**	-0.677** -0.502**	-0.630** -0.516**	-0.625** -0.338**	-0.428** -0.561**	0.132** 0.030	0.493** 0.251**	1.000
PPLEL	0.153** 0.061**	-0.071** -0.012	-0.003 *0.009*	0.114** 0.068**	-0.797** -0.786**	-0.615** -0.657**	-0.699** -0.672**	-0.672** -0.640**	0.357** 0.376**	0.068** 0.054**	0.557** 0.478**

*Significant at the 0.05 level (2-tailed); **Significant at the 0.01 level (2-tailed).

^§^Note: ASM (age-standard mortality rates); RF (rainfall); MinT (minimum temperature); MaxT (max temperature); IRSAD (Index of Relative Socio-economic Advantage and Disadvantage); IRSD (Index of Relative Socio-economic Advantage and Disadvantage); IER (Index of Economic Resources); IEO (Index of Education and Occupation); PIP (proportion of Indigenous population); UER (unemployment rate); PPLII (proportion of population with low individual income); PPLEL (proportion of population with low educational level).

In the assessment of multicollinearity between socio-environmental variables, we found that some SEIFA indexes (e.g., IRSAD and IER) were highly correlated (r = 0.90). Thus, IRSD index was used to represent SEIFA in this study because of its strongest association with suicide across four SEIFA indexes. In addition, MinT and MaxT were also highly correlated (r = 0.85), and therefore, we use MaxT and MinT in separate models.

### Multivariable analysis

**Figure 10:**
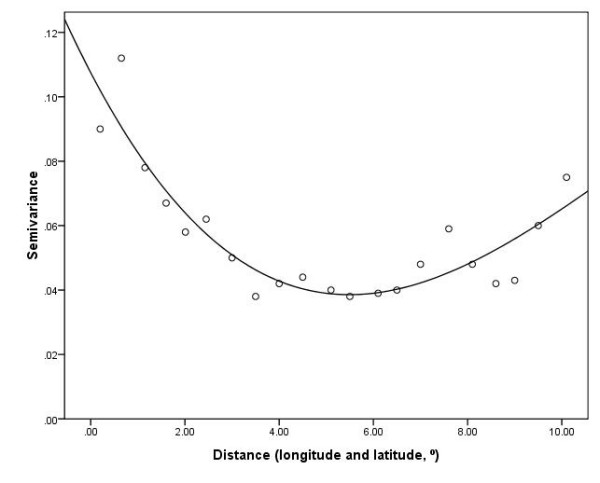
Semivariogram of model residuals

Multivariable GEE models were undertaken to examine the possible impact of climate variables, SEIFA and demographic factors on suicide mortality rate. We used the semivariance to measure the degree of spatial autocorrelation of model residuals. Figure 10 shows that there was no increased semivariance of residuals when the distance of LGAs increased. Thus it suggests that the GEE model fitted the data well as there was little spatial autocorrelation of residuals in this study.

Table 4 shows the associations between socio-environmental variables and suicide mortality by gender. In males, MaxT, PIP and PPLII were significantly and positively associated with suicide in male population. RF, IRSD, UER and PPLEL were not significantly associated with male suicide. In females, PIP and UER were statistically significantly associated with suicide, but there was no significant association for other variables.

**Table 4:** Regression of socio-environmental determinants of suicide for both male (upper value) and females (lower value, italics)*

Variables	β	SE	RR (95% CI)	P-value
RF (mm)	0.090 *0.114*	0.073 *0.291*	1.09 (0.95 -- 1.26) *1.12(0.63 -- 1.98)*	0.220 *0.696*
MaxT (°C)	0.213 *-0.072*	0.088 *0.248*	1.24 (1.04 -- 1.47) *0.93(0.57 -- 1.51)*	0.016 *0.773*
IRSD	-0.017 *0.052*	0.026 *0.090*	0.98 (0.94 -- 1.03) *1.05 (0.88 -- 1.26)*	0.483 *0.565*
PIP (%)	0.065 *0.209*	0.030 *0.094*	1.07 (1.01 -- 1.13) *1.23 (1.03 -- 1.48)*	0.029 *0.026*
UER (%)	0.077 *0.087*	0.101 *0.042*	1.08 (0.89 -- 1.32) *1.09 (1.01 -- 1.18)*	0.446 *0.036*
PPLII (%)	0.373 *-0.176*	0.086 *0.137*	1.45 (1.23 -- 1.72) *0.84 (0.64 -- 1.10)*	0.000 *0.198*
PPLEL (%)	-0.139 *0.168*	0.117 *0.106*	0.87 (0.69 -- 1.09) *1.18 (0.96 -- 1.46)*	0.234 *0.115*

*Note: RR (relative risk); CI (confidential interval); RF (rainfall); MaxT (max temperature); IRSD (Index of Relative Socio-economic Advantage and Disadvantage); PIP (proportion of Indigenous population); UER (unemployment rate); PPLII (proportion of population with low individual income); PPLEL (proportion of population with low educational level).

## Discussion

This study examined the relationship between socio-environmental factors and suicide using GIS and spatiotemporal analysis approaches. A range of climate, socioeconomic and demographic determinants were included in this quantitative analysis.

The results of this study indicate some key socio-environmental predictors of suicide at the LGA level. The preliminary spatiotemporal analyses show that far north Queensland had the highest suicide mortality, while the south-western areas had the lowest mortality rate in all the seasons. MaxT, PPLII and PIP were positively associated with total and male suicide. UER had a positive association with total and female suicide. RF had a significant and positive association with total suicide only. However, no significant association was found for SEIFA and PPLEL.

Some of the previous studies found that rainfall was negatively associated with suicide [4,32], while some other studies showed that this association was very weak [33,34]. Persistent rainfall deficiency results in drought, which causes reduction of crops in rural areas and adds financial burden to local residents, especially farmers [32,35]. In rural areas, farmers and other residents usually have less social support than urban residents, and this situation can get worse due to drought [36]. All these add stress, anxiety and mental health problems among the rural population which will eventually lead to suicidal behaviours and even suicide. However, this study only covered 5-year rainfall and suicide data, so it is difficult to determine the long term effect of rainfall on suicide. Another explanation for this discrepancy is that Queensland is in tropical and subtropical areas with much rainfall in general, especially in coastal areas. Even during drought periods, rainfall in Queensland is still much higher than other states in Australia.

In this study, higher MaxT was accompanied with increased suicide mortality at a LGA level. This finding corroborates previous reports [6,37,38]. For instance, some studies discovered that higher temperature can lead to decreased availability of tryptophan in human body, one of the 20 standard amino acids, then the volume of 5-Hydroxyindoleacetic acid (5-HIAA) synthesized from tryptophan greatly reduced [39]. As 5-HIAA can reduce depression among humans [40], therefore, the reduced 5-HIAA indirectly caused by high temperature leads to more depression and other mental health problems among population, even suicidal behaviours. We also examined the association between minimum temperature and suicide in the GEE model, but the association was very weak.

This study demonstrates a general trend that LGAs with higher PIP had higher rates of suicide. As most of the Indigenous population are located in rural areas, these communities often have lower SES and less opportunities of healthcare, including mental health services. The rapid social change in Australia may also affect the Indigenous communities, with more unhealthy behaviours such as excessive alcohol use and family violence [41]. The environmental injustice in this study should not be ignored. Some activities (e.g., construction of water systems, land use, and management of organizations) may cause cultural, environmental and economic risks and hazards among the local communities, especially in the areas with low SES and high proportion of Indigenous population [42,43]. The above factors contribute to the higher suicide occurrence and deaths in communities with a high proportion of Aboriginal and Torres Strait Islanders in Queensland [44]. Other studies in the United States also indicate that suicide mortality rates were higher in the areas with higher proportion of Indigenous population than in the other areas [45-47]. These studies also discovered that suicide is associated with harsh environmental and social conditions.

Increased unemployment rate directly reduces individual and family income, and thus can cause the financial burden and result in anxiety and stress among family members, especially for a less skilled population. These may increase the risk of mental health problems and suicidal behaviours. This can explain why unemployment had an adverse impact on suicide. Previous studies also discovered that higher unemployment rate can enhance the risk of suicide behaviour and suicide [48-50]. In this study, unemployment had more significant impact on female suicide than male suicide. In recent years, more females participated in labour force than before, thus more females would experience unemployment as a consequence [51]. A study in Portugal also indicated that female suicide increased, as women play more important roles in the socioeconomic status and the stress of unemployment on them was more prominent than before [52].

Previous studies have indicated that higher socioeconomic status (SES) areas usually have lower suicide mortality [10,11], as high SES areas usually have higher employment rates, increased income and more accesses to training and education, compared with low SES areas [53]. In this study, we did not find a significant association between SEIFA and suicide, which may be due to a short time series dataset (5 years) and the use of a snapshot measure of SEIFA (i.e., disadvantage index in 2001).

Some studies indicate that the population with low income had higher suicide rate [54,55]. Generally, rural areas have higher proportion of population with low income, while healthcare (including mental health care) facilities are less developed and less accessible than urban areas. This can lead to increased mental health problems, even suicidal behaviours, among the local population. The results of this study are consistent with previous studies.

Some studies conducted in temperate areas like Brazil [56] and Italy [57] observed a peak of suicide in late spring and early summer. In this study, there were suicide peaks in August and October between 1999 and 2003. More suicides occurred in summer than other seasons. The results in this study were not completely consistent with previous studies, partly because all the LGAs of Queensland are in tropical and subtropical zones, and the four seasons are not evident in many places, especially in the north Queensland.

This study has several strengths. Firstly, this is the first study to examine an association between a wide range of socio-environmental factors and suicide at a LGA level in Queensland. Secondly, this study used a comprehensive spatial dataset, GIS and a range of quantitative analytical methods to compare the differences of socio-environmental impact on suicide over time and space. Thirdly, this study examined how socio-environmental factors influence the likelihood of suicide after taking into account a range of confounding factors, including gender, population size and SEIFA at the LGA level. Finally, the results of this study may have implications in public health policy making and implementation of suicide prevention intervention.

The limitations of this study should also be acknowledged. Firstly, the time series data set for analysis is short, compared with other studies [32,37,57]. Secondly, climate condition varies in different zones within each LGA, especially those covering large areas. So it is difficult to actually determine the climate condition in the geographical spot of each suicide death. Thirdly, the SEIFA index and demographic data at the LGA level were only based on 2001 Population Census, so it cannot reflect any changes in socioeconomic and demographic features during the whole study period. Thus the results of this study should be interpreted cautiously. Finally, this study only included several socio-environmental variables (i.e., rainfall, temperature, SEIFA, demographic variables), while other factors like personal and family history of mental health and psychiatric problems [58,59], local health service facilities [60], nutrition [61,62], religion [63,64], alcohol and drug use [65-67] may also influence mental health status and suicidal behaviours. However, the information on these variables was unavailable in this study.

The impact of socio-environmental change on mental health has drawn much attention. On the one hand, the current global financial crisis is likely to deepen, and it will almost certainly have negative effects on the trend of suicide. On the other hand, as climate change continues, the frequency, intensity and duration of weather extremes (e.g., flood, drought and cyclone) are likely to increase in the coming decade, it may also lead to the increase in suicide. Thus it is vital to strengthen surveillance system on weather extremes (e.g., high temperature) and social changes (e.g., unemployment) as well as the impacts of these changes on mental health [68-70]. Governmental officials, epidemiologists, psychiatrists, environmental health workers, economists, meteorologists and community leaders should work together to design, develop and implement effective suicide prevention and control strategies through an integrated and systematic approach.

## Conclusion

In this study, we discovered that suicide ASM varied between LGAs by gender. Maximum temperature, the proportion of Indigenous population and unemployment rate appeared to be major determinants of suicide at a LGA level in Queensland. Other factors, such as rainfall, education and income level, had no significant association with suicide at a LGA level, during the period 1999-2003. These findings may have implications in planning and implementing population-based suicide interventions.

## List of abbreviations

ABS: (Australian Bureau of Statistics); ASM: (age-adjusted standardized mortality); CI: (confidential interval); GIS: (geographical information system); GEE: (generalized estimating equation); IRSAD: (Index of Relative Socio-economic Advantage and Disadvantage); IRSD: (Index of Relative Socio-economic Advantage and Disadvantage); IEO: (Index of Education and Occupation); IER: (Index of Economic Resources); LGA: (Local Governmental area); MinT: (minimum temperature); MaxT: (max temperature); PIP: (proportion of Indigenous population); PPLII: (proportion of population with low individual income); PPLEL: (proportion of population with low educational level); RF: (rainfall); RR: (relative risk); SEIFA: (Socioeconomic Indexes for Areas); SLA: (statistical local area); UER: (unemployment rate);

## Competing interests

The authors declare that they have no competing interests.

## Authors' contributions

XQ designed the study, implemented all statistical analyses and drafted the manuscript. ST conceptualised the idea and revised the study protocol, especially the research design and data analysis. WH contributed to statistical analyses and interpretation of the results. All the authors contributed to the preparation of the final manuscript and approved the submission.
